# Multiple Histone Lysine Methyltransferases Are Required for the Establishment and Maintenance of HIV-1 Latency

**DOI:** 10.1128/mBio.00133-17

**Published:** 2017-02-28

**Authors:** Kien Nguyen, Biswajit Das, Curtis Dobrowolski, Jonathan Karn

**Affiliations:** Department of Molecular Biology and Microbiology, Case Western Reserve University School of Medicine, Cleveland, Ohio, USA; Columbia University

## Abstract

We showed previously that the histone lysine methyltransferase (HKMT) H3K27me3 (EZH2) is the catalytic subunit of Polycomb repressive complex 2 (PRC2) and is required for the maintenance of HIV-1 latency in Jurkat T cells. Here we show, by using chromatin immunoprecipitation experiments, that both PRC2 and euchromatic histone-lysine *N*-methyltransferase 2 (EHMT2), the G9a H3K9me2-3 methyltransferase, are highly enriched at the proviral 5′ long terminal repeat (LTR) and rapidly displaced upon proviral reactivation. Clustered regularly interspaced short palindromic repeat(s) (CRISPR)-mediated knockout of EZH2 caused depletion of both EZH2 and EHMT2, but CRISPR-mediated knockout of EHMT2 was selective for EHMT2, consistent with the failure of EHMT2 knockouts to induce latent proviruses in this system. Either (i) knockout of methyltransferase by short hairpin RNA in Jurkat T cells prior to HIV-1 infection or (ii) inhibition of the enzymes with drugs significantly reduced the levels of the resulting silenced viruses, demonstrating that both enzymes are required to establish latency. To our surprise, inhibition of EZH2 (by GSK-343 or EPZ-6438) or inhibition of EHMT2 (by UNC-0638) in the Th17 primary cell model of HIV latency or resting memory T cells isolated from HIV-1-infected patients receiving highly active antiretroviral therapy, was sufficient to induce the reactivation of latent proviruses. The methyltransferase inhibitors showed synergy with interleukin-15 and suberanilohydroxamic acid. We conclude that both PRC2 and EHMT2 are required for the establishment and maintenance of HIV-1 proviral silencing in primary cells. Furthermore, EZH2 inhibitors such as GSK-343 and EPZ-6438 and the EHMT2 inhibitor UNC-0638 are strong candidates for use as latency-reversing agents in clinical studies.

## IMPORTANCE

Highly active antiretroviral therapy (HAART) reduces the circulating virus to undetectable levels. Although patients adhering to the HAART regimen have minimal viremia, HIV persists because of the existence of latent but replication-competent proviruses in a very small population of resting memory CD4^+^ T cells (~1 in 10^6^ cells). Latency remains the major obstacle to a functional cure for HIV infection, since the persistent reservoir almost invariably rebounds within 2 to 8 weeks when HAART is interrupted. In latently infected cells, the HIV genome is stably integrated into the host chromosome but transcriptionally repressed because of epigenetic silencing mechanisms. We demonstrate here that multiple histone lysine methyltransferases play a critical role in both the establishment and maintenance of proviral silencing in cells obtained from well-suppressed patients. Drugs that inhibit these enzymes are available from oncology applications and may find a use in reversing latency as part of a reservoir reduction strategy.

## INTRODUCTION

Silenced, or latent, proviruses produce minimal amounts of viral RNA and proteins, allowing them to persist in the face of highly active antiretroviral therapy (HAART) and to evade immune responses. Latency has emerged as a major obstacle to a functional cure for HIV infection, since the persistent reservoir almost invariably rebounds within a few weeks after treatment interruption (1, 2). The latent reservoir is composed of a transcriptionally silent population of viruses that reside primarily in memory CD4^+^ T cells and, in some patients, microglial cells in the central nervous system (3, 4).

Even though latently infected cells are found at a frequency of <10^6^ in peripheral T cells from well-suppressed HAART-treated patients (5), elimination of this tiny population of cells is challenging, since the latent reservoir is very stable, with an apparent half-life of 44 months in the presence of ART (5, 6). Recent studies of proviral integration sites have provided strong evidence for the clonal expansion of specific proviruses (7, 8). These data suggest strongly that the reservoir is in a pseudosteady state with persistent low rates of viral reactivation and cell death counterbalanced by intermittent viremia (9) and homeostatic expansion of latent clones (7, 8, 10).

One of the most promising avenues for eradicating the latent reservoir is the “shock and kill” strategy, which involves reactivation of latent HIV-1 by latency-reversing agents (LRAs) in the presence of HAART (11, 12) to make latently infected cells visible to immunological surveillance. Clinical and *ex vivo* studies suggest that viral reactivation alone is unlikely to achieve eradication (13, 14) because of insufficient viral cytopathic effects and defective cytotoxic T lymphocyte responses (15). Therefore, HIV eradication will require not only efficient reactivation of proviruses but also a coupled and targeted immunotherapeutic strategy.

A complex combination of cellular events that suppress both the initiation of HIV transcription and its productive elongation is required for the establishment of latent proviruses (12, 16). In addition, epigenetic silencing events are critical to maintain proviral latency (4, 16). The majority of integrated proviruses (~85%) in patients are defective, and within the population of genetically intact proviruses, 10 to 20% are refractory to potent T-cell activation stimuli such as activation of the T-cell receptor (TCR) or treatment with phorbol myristate acetate/phytohemagglutinin (17). Single LRAs that have been studied clinically, such as histone deacetylase (HDAC) inhibitors (i.e., suberanilohydroxamic acid [SAHA], panobinostat, and romidepsin) are generally weak inducers of latent proviruses (18, 19), suggesting that combinations of agents will be required to efficiently induce latent proviruses (20–22). An important part of the HIV eradication effect is therefore directed at identifying new mechanisms that control HIV latency and validating safe and effective combinations of agents that can activate the majority of latent proviruses.

We showed previously that EZH2, the H3K27 histone lysine methyltransferase (HKMT) component of Polycomb repressive complex 2 (PRC2), plays a critical role in controlling HIV latency in Jurkat T-cell models of latency (23, 24). Other groups have implicated additional histone methyltransferases, including SUV39H1 and euchromatic histone-lysine *N*-methyltransferase 2 (EHMT2), in the control of latency (24–26). This discrepancy could be due to variable responses of HIV proviruses to LRAs because of heterogeneity in the epigenetic blocks imposed on proviruses, clonal variations, and cell line-specific responses. In fact, even single clones show variable responses to activating agents that are associated with variations in histone methylation marks (24).

Since it remains an open question whether each of the H3K27 and H3K9 methyltransferases plays a distinct role in controlling HIV transcription, we compared the roles of PRC2 and EHMT2 in the maintenance and establishment of HIV latency in the Jurkat cell model, a Th17 primary cell model of HIV latency, and in patient cells. In Jurkat cells both HKMTs are required to silence HIV proviruses but PRC2 is distinctive because it controls the major rate-limiting step restricting proviral reactivation. In contrast, in both the primary cell models and patient cells, PRC2 and EHMT2 are each required to establish and maintain latency.

## RESULTS

### Identification of HKMTs required to maintain silencing of HIV-1 transcription in T cells by shRNA library screening.

To identify epigenetic regulators that are required to maintain HIV latency, we performed genome-wide short hairpin RNA (shRNA) screening by using a comprehensive shRNA library, combined with system biology classifications of the “hits.” Two latently infected Jurkat T-cell lines (2D10 and E4 cells) (24, 27, 28) were superinfected with a synthetic shRNA library (Cellecta, Inc., Mountain View, CA) containing a total of 82,500 shRNAs targeting 15,439 mRNA sequences. Green fluorescent protein-positive (GFP^+^) cells carrying reactivated proviruses were then purified by sorting twice and the shRNA sequences were identified by next-generation sequencing. The protocol results in the clonal enrichment of cells carrying shRNAs that stably reverse HIV latency. Thus, the number of sequence reads obtained for any given shRNA is proportional to the number of enriched cells carrying the shRNA and provides a measure of the potency of the shRNA.

As shown in Table 1, the screening confirmed that PRC2 contributes to silencing in these cells. The ranking of the complexes and relative potency of shRNAs directed against individual subunits in both complexes was very consistent between the 2D10 and E4 cells, with hits in the top 10% considered to be significant. Among the eight different subunits of PRC2, EZH2, SUZ12, and JARID2 ranked in the top 10% in the E4 cell screening and EZH2, EZH1, RBBP7, SUZ12, and JARID2 ranked in the top 10% in the 2D10 cell screening.

**TABLE 1:** Enrichment of histone methyltransferase complexes in shRNA screening^a^

Protein	E4		2D10	
	Rank	Percentile	Rank	Percentile
PRC2				
EED	10,615	38.65	4,704	31.65
EZH2	2,654	9.66	518	3.48
EZH1	7,701	28.04	90	3.35
RBBP4	4,920	17.91	3,364	22.63
RBBP7	3,370	12.27	444	2.98
SUZ12	1,820	6.62	840	5.65
AEBP2	2,768	10.14	2,538	11.74
JARID 2	2,688	9.77	1,744	6.99
Avg		16.63		10.72
PRC1				
BMI1	4,013	14.61	830	5.58
CBX2	567	2.06	757	5.09
CBX4	481	1.75	2,538	17.07
CBX8	3,302	12.09	5,478	25.34
HSPA1A	2,430	8.84	2,804	18.86
PHC1	5,701	20.75	4,215	28.36
PHC2	679	2.48	3,447	15.94
PHC3	2,434	8.911	1,172	5.42
RING1	14,235	51.83	10,885	73.25
RNF2	1,916	6.97	1,372	9.23
SCMH1	10,905	39.95	7,812	36.14
SMARCA5	2,648	9.63	5,743	23.03
YY1	8,428	30.64	6,074	40.87
Avg		16.20		23.40
SUV39H1-HP1 complex				
CBX5	64	0.23	195	1.31
MBD1	4,055	14.76	86	0.57
SUV39H1	4,258	15.50	472	3.17
Avg		10.16		1.68
CtBP complex				
KDM1B	7,103	25.83	13,817	55.40
CTBP1	472	1.71	5,378	36.19
CTBP2	438	1.59	1,720	11.57
EHMT1	13,002	47.34	2,613	17.58
EHMT2	3,713	13.51	2,638	17.75
HDAC1	14,496	52.78	1,059	7.12
HDAC2	5,973	21.74	10,002	67.30
RCOR1	9,550	34.98	2,804	12.97
ZEB1	2,012	7.31	3,505	14.05
Avg		22.98		26.66

^a^ The latently infected Jurkat cell lines E4 (wild-type Tat) and 2D10 (H13L Tat) (28) were superinfected with a synthetic shRNA library (Cellecta, Inc., Mountain View, CA) containing a total of 82,500 shRNAs targeting 15,439 mRNA sequences in three modules. The data shown are almost exclusively from module 1, which is focused on transcriptional control. d2EGFP^+^ cells carrying reactivated proviruses were purified by sorting twice, and the shRNA sequences were identified by next-generation sequencing. The sorting resulted in clonal outgrowth of cells where latency had been reversed, and this was reflected in a skewed distribution of reads. In the case of the E4 cells, the reads ranged from 19,619 to 1 with 27,464 shRNAs identified. For 2D10 cells, the reads ranged from 843,188 to 1 with 14,860 shRNAs identified. shRNAs were ranked in read order with the highest rank being 1.

Subunits of PRC1 and the CtBP complex, carrying the H3K9me1,2 histone methyltransferase EHMT2 (G9a), were also highly enriched in the screening. SUV39H1-HP1, an H3K9me2,3 histone methyltransferase, appeared to be a potent “hit” in 2D10 cells but was only in the 15% range in E4 cells. Thus, these unbiased screening results strongly indicate that the histone methylation machinery is required for HIV silencing and latency.

### EZH2, but not EHMT2, is required to maintain HIV latency in Jurkat cells.

**FIG 1:**
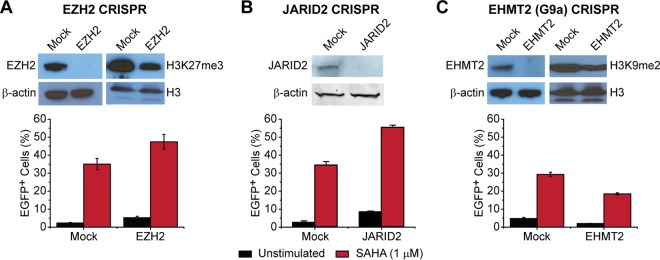
CRISPR-mediated depletion of EZH2 or JARID2 reactivates latent HIV-1 in E4 cells. Panels: A, EZH2-disrupted cells; B, JARID2-disrupted cells; C, EHMT2 (G9a)-disrupted cells. (Top) Western blot assays showing histone methyltransferases and methylated histones. (Bottom) Quantification of HIV-1 reactivation. E4 cells were infected with CRISPR-Cas9-expressing viruses targeting the proteins indicated. Reactivation of HIV-1 by stimulation with SAHA (1 μM) overnight was measured 7 days postinfection by FACS analysis. Error bars represent the SEM of three separate experiments.

To investigate the roles of EZH2 and EHMT2 in the reactivation of latent proviruses in E4 cell, we knocked out the genes by using clustered regularly interspaced short palindromic repeat(s) (CRISPR)-Cas9 (Fig. 1). Concomitant with the depletion of EZH2 and EHMT2, we observed significant reductions in the levels of H3K27me3 and H3K9me2, respectively. However, only the depletion of EZH2 induced a weak spontaneous reactivation of latent proviruses. The depletion of EZH2 also enhanced the reactivation of latent proviruses by stimulation with 1 μM SAHA. When viruses were reactivated by SAHA, approximately 30% of them were reactivated, while approximately 50% of them were reactivated under these conditions when EZH2 was ablated. In contrast, no reactivation of latent proviruses was observed when EHMT2 was knocked out in E4 cells and there were no additive effects observed in the presence of SAHA (Fig. 1).

**FIG 2:**
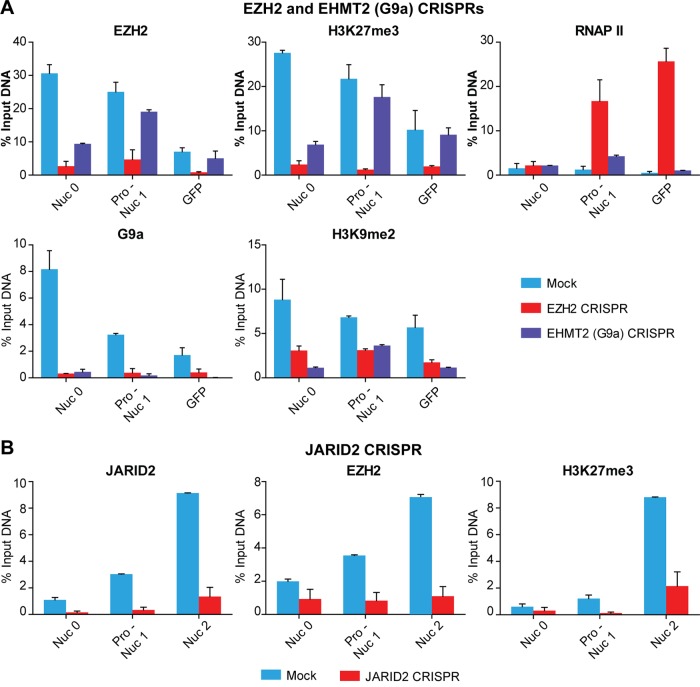
Distribution of histone methyltransferases and methylated histones on HIV proviruses following CRISPR disruption. (A) EZH2 and EHMT2 disruption. (B) JARID2 disruption. ChIP assays were performed with E4 cells infected with the CRISPRs indicated by using primers to the HIV LTR and downstream regions (27). HIV DNA levels were calculated as percentages of the input. Error bars represent the SEM of three separate real-time PCR measurements.

Chromatin immunoprecipitation (ChIP) assays showed that these proteins were highly enriched in the HIV-1 genome in E4 cells infected with mock CRISPR-Cas9 viruses and substantially removed from the HIV-1 provirus when the genes were disrupted (Fig. 2). Together with the removal of EZH2 and EHMT2, there were substantial reductions in the levels of H3K27me3 and H3K9me2 at the HIV-1 long terminal repeat (LTR). Interestingly, knockout of EZH2 resulted in the depletion of both H3K27me3 and H3K9me2, while knockout of G9a only caused a reduction in the level of H3K9me2. Furthermore, enhanced accumulation of RNA polymerase (RNAP) II at the promoter-proximal pause site was observed only when EZH2 was knocked out (24).

These results are consistent with shRNA experiments used to reduce the expression of each of PRC2 subunits, EHMT2 (G9a), the H3K9-specific demethylase KDM1 (LSD1), and SUV39H1 (see Fig. S1 and S2 in the supplemental material). ChIP assays show that all of the components of the H3K27 and H3K9 silencing machinery are present at the LTRs of latent proviruses and removed after activation with tumor necrosis factor alpha (TNF-α) (see Fig. S3). KDM1 (LSD1), an H3K9 demethylase that, together with EHMT2, forms part of the CoREST and CTIP2 repressor complexes, was also present at the LTR and declined after TNF-α activation (see Fig. S3).

10.1128/mBio.00133-17.1 FIG S1  PRC2 represses HIV-1 transcription in E4 cells. (A) Representative FACS analysis measuring HIV-1 (d2EGFP) expression in cells left untreated or treated with SAHA (1 µM) overnight. E4 cells were infected with shRNAs targeting different subunits of PRC2. Reactivation of HIV by stimulation with SAHA (1 µM) was measured 7 days after infection. Knockdown of PRC2 subunits by shRNAs not only spontaneously induce proviral reactivation but also potentiated the reactivation of HIV-1 by stimulation with SAHA. (B) Western blot assays showing the expression of the different subunits of PRC2 in E4 cells expressing shRNAs against the proteins indicated. shRNA expression resulted in >50% depletion of the PRC2 proteins indicated. Fifty micrograms of total cell lysate or nuclear extract was loaded. (C) Quantification of HIV-1 reactivation in E4 cells expressing shRNAs to PRC2. Reactivation of HIV by TCR stimulation (anti-CD3 antibody [0.125 µg/ml] plus anti-CD28 antibody [1 µg/ml]), SAHA (1 µM), or TNF-α (1 ng/ml) overnight was measured by FACS analysis 7 days after infection. Error bars represent the SEM of three separate experiments. Note that knockdown of PRC2 in E4 cells also sensitized proviruses to reactivation by each stimulus. (D) ChIP assay measuring the enrichment of EZH2, EED, or SUZ12 at the HIV-1 LTR in E4 cells under untreated or reactivated conditions. E4 cells were treated with TNF-α (10 ng/ml) for 30 min. Download FIG S1, TIF file, 3.5 MB. Copyright © 2017 Nguyen et al. 2017 Nguyen et al. This content is distributed under the terms of the Creative Commons Attribution 4.0 International license.

10.1128/mBio.00133-17.2 FIG S2  Knockdown of EHMT2 (G9a), KDM1 (LSD1), and SUV39H1 does not reactivate HIV-1 in E4 cells. FACS experiments monitored the reactivation of HIV-1 in E4 cells infected with lentiviral vectors expressing EHMT2 (G9a), KDM1 (LSD1), and SUV39H1 shRNAs under untreated (A) or SAHA-stimulated (B) conditions. E4 cells were infected with the shRNA vectors indicated, selected in puromycin (2 µg/ml)-supplemented medium for 4 days, and then subjected to SAHA treatment overnight. d2EGFP expression in the cells was measured by FACS. Note that H3K9 methyltransferases do not play an important role in the control of HIV-1 latency in E4 cells. Download FIG S2, TIF file, 1.7 MB. Copyright © 2017 Nguyen et al. 2017 Nguyen et al. This content is distributed under the terms of the Creative Commons Attribution 4.0 International license.

10.1128/mBio.00133-17.3 FIG S3  Proteins of the PRC2 and H3K9 methylation machinery are removed from the HIV-1 provirus upon reactivation. ChIP assays were performed with unstimulated (light blue bars) and TNF-α-stimulated (dark blue bars) E4 cells (30 min). E4 cells were treated with 10 ng/ml TNF-α for 30 min. The enrichment of HIV-1 DNA was analyzed with several primers surrounding the HIV-1 promoter. Antibodies against the proteins indicated, H3K27me3, H3K9me2, and H3K9me2-3 were used. Error bars represent the SEM of three separate real-time PCR measurements. Note that all of the core subunits of PRC2, EZH2, EED, and SUZ12, were present at the HIV-1 LTR together with a high level of H3K27me3 marks. Upon TNF-α reactivation, each of the PRC2 subunits was removed and the level of H3K27me3 was also dramatically decreased. Thus, PRC2 is deposited at latent HIV-1 proviruses and functions as a repressive complex. A significant enrichment of G9a or SUV39H1 was also detected at the 5′ LTR of latent viruses and displaced upon TNF-α treatment. The levels of the H3K9me2 and H3K9me2-3 epigenetic silencing marks declined concomitantly. KDM1 (LSD1), an H3K9 demethylase that, together with G9a, forms part of the CoREST and CTIP2 repressor complexes, was also present at the LTR and declined after TNF-α activation. Download FIG S3, TIF file, 1.2 MB. Copyright © 2017 Nguyen et al. 2017 Nguyen et al. This content is distributed under the terms of the Creative Commons Attribution 4.0 International license.

### JARID2 mediates PRC2 recruitment to latent HIV-1 proviruses.

Many studies suggest that in mammalian systems, JARID2 mediates the recruitment of PRC2 to its target genes (29). In our shRNA screenings, shRNAs targeting JARID2 were highly ranked (Table 1). As shown in Fig. 1, CRISPR-mediated knockout of JARID2 expression in E4 cells resulted in enhanced spontaneous reactivation of HIV-1 and sensitized latent proviruses to additional stimulation by SAHA. The level of proviral reactivation acquired from JARID2 knockout was comparable to that observed when we knocked out EZH2.

When we performed ChIP assays of JARID2-disrupted E4 cells, we observed a significant reduction in the level of JARID2 association with the HIV LTR compared to that of E4 cells infected with mock CRISPR-Cas9 viruses. Concomitantly with reductions in the levels of JARID2 along different positions of the HIV-1 genome, parallel reductions in the levels of EZH2 and H3K27me3 were also detected in exactly the same regions (Fig. 2B). Therefore, we concluded that JARID2 plays a critical role in the recruitment of PRC2 to the HIV-1 LTR.

### PRC2 is required for “silent integration” of HIV-1 in Jurkat T cells.

**FIG 3:**
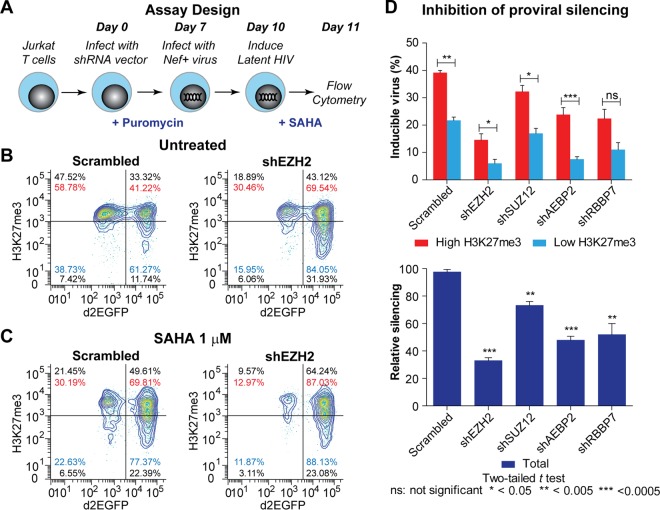
PRC2 is required for silent integration of HIV-1 in Jurkat T cells. (A) Experimental scheme. (B) Representative flow data measuring levels of H3K27me3 and HIV infection in cells expressing EZH2 or scrambled shRNA in untreated cells. (C) Cells treated with 1 µM SAHA. Black, cell counts per quadrant; red, distribution of GFP^+^ cells in high-H3K27me3 gate; blue, distribution of GFP^+^ cells in low-H3K27me3 gate. (D) Relative silencing of HIV-1 from cells treated with shRNAs targeting different subunits of PRC2. (Top) Inducible virus in high- and low-H3K27me3 populations. (Bottom) Relative silencing after knockdown of each of the PRC2 subunits. Error bars represent the SEM of three separate experiments.

Infection of cells by HIV-1 results in a high fraction of “silent integration” events where the virus integrates into active regions of the chromatin but is largely transcriptionally inactive (30). To determine whether PRC2 is also required for these silent integration events, we used the experimental design illustrated in Fig. 3A. Briefly, we knocked down PRC2 proteins in Jurkat E6 cells with shRNAs and then superinfected the cells with vesicular stomatitis virus G (VSV-G)-pseudotyped HIV-1. Three days after HIV-1 superinfection, intracellular levels of H3K27me3 marks were measured by fluorescence-activated cell sorting (FACS), while HIV infection efficiency was monitored by d2EGFP expression. We subsequently treated infected cells with SAHA to reactivate silent viruses.

To analyze the results, we gated cells into two populations, high and low H3K27me3. We calculated the percentage of d2EGFP expression in each population. The values are shown in red for the high-H3K27me3 population and in blue for the H3K27m3 low population in Fig. 3B and C. For clarity, only flow data from the infection conditions used with cells expressing scrambled and EZH2 shRNAs are shown. For data from cells expressing other PRC2-targeting shRNAs, see Fig. S4 and S5.

10.1128/mBio.00133-17.4 FIG S4  Knockdown of PRC2 subunits reduces intracellular H3K27 trimethylation levels in Jurkat E6 T cells. Cells were spotted onto poly-l-lysine-coated coverslips, fixed with 4% formaldehyde, and then permeabilized with 0.1% Triton X-100. Permeabilized cells were blocked with 5% normal donkey serum. Cells were stained with anti-histone H3 trimethyl K27 mouse MAb (1:2,000 dilution; 6002; Abcam, Inc.) for 30 min and then with an Alexa Fluor 647-conjugated AffiniPure goat anti-mouse IgG (H+L) secondary antibody (1:2,000 dilution; 115-605-003; Jackson ImmunoResearch) for 20 min. 4',6-Diamidino-2-phenylindole (DAPI) staining was performed for 2 min at room temperature. The intensity of H3K27me3 staining was significantly lower in PRC2-depleted cells than in cells expressing scrambled shRNA, consistent with the flow cytometry data in Fig. S5. Download FIG S4, TIF file, 5.5 MB. Copyright © 2017 Nguyen et al. 2017 Nguyen et al. This content is distributed under the terms of the Creative Commons Attribution 4.0 International license.

10.1128/mBio.00133-17.5 FIG S5  Knockdown of PRC2 reduces the portion of latent infection of HIV-1 in Jurkat T cells. (A) Two-dimensional histogram measuring the global levels of H3K27me3 and monitoring HIV-1 infection in the cells 3 days after HIV-1 superinfection. Jurkat T cells were first infected with PRC2 shRNA viruses and then superinfected with VSV-G-pseudotyped HIV-1. The *x* axis represents levels of expression of d2EGFP, a marker of HIV-1 reactivation. The *y* axis represents the global level of H3K27me3, which is measured by staining with anti-H3K27me3 antibody. The gates for H3K27me3 levels were set on the basis of the fluorescence intensity of cells stained only with the rabbit isotype MAb labeled with Alexa Fluor 647. Black, cell counts per quadrant; red, distribution of GFP^+^ cells in high-H3K27me3 gate; blue, distribution of GFP^+^ cells in low-H3K27me3 gate. (B) FACS analysis measuring global levels of H3K27me3 in PRC2 knockdown cells. Download FIG S5, TIF file, 4.5 MB. Copyright © 2017 Nguyen et al. 2017 Nguyen et al. This content is distributed under the terms of the Creative Commons Attribution 4.0 International license.

If EZH2 (or any other epigenetic regulator) is required for establishing silent integration events, then we would expect to see an enhancement of GFP^+^ cells in the initial infection and a reduction in the number of GFP^+^ cells after treatment with 1 μM SAHA for 18 h. In cells expressing EZH2 shRNA (Fig. 3B and C), SAHA treatment caused only a slight increase in d2EGFP expression (from 84 to 87%) in the low-H3K27me3 population, while the same treatment resulted in a 17% increase in d2EGFP expression (from 69 to 86%) in the high-H3K27me3 population. Similar results were seen in cells expressing shRNAs targeting other subunits of PRC2 (see Fig. S5). These differences in viral responsiveness between the two cell populations, normalized to the total number of virus-infected cells in each population (the inducible virus percentage), were statistically significant for all of the PRC2 subunits (Fig. 3D, top). The total number of virus-infected cells in each population was the total number of d2EGFP^+^ cells in the same population following SAHA treatment. For example, for the EZH2 subunit, the percentages of inducible viruses would be 20.1% (17%/87%) and 4.6% (4%/88%) for the high- and low-H3K27me3 populations of cells, respectively.

These data were also used to calculate the relative proportion of silenced proviruses in the scrambled and PRC2-targeted-shRNA-expressing Jurkat cells (regardless of H3K27me3 levels) (Fig. 3D). For each subunit of PRC2, we first calculated the total percentage of silent viruses, which was the percent difference in the number of d2EGFP^+^ cells between SAHA-stimulated and untreated conditions. The relative silencing value was then calculated by dividing the percentage of silent viruses observed under knockdown conditions by that from the scrambled control. For example, as shown in Fig. 3B and C, for the EZH2 subunit, the total percentages of d2EGFP^+^ cells before and after SAHA treatment were 75.05% and 87.32%, respectively. The percentage of silenced viruses from the EZH2 knockdown condition was therefore 14% ([87.32 to 75.05]/87.32). Similarly, the percentage of silenced viruses from the scrambled control was 37.4% ([72 to 45.06]/72).

The relative silencing value (Fig. 3D) was calculated by dividing the normalized total percentage of silent viruses from the PRC2 knockdowns by that from the scrambled control. The relative silencing values for EZH2 knockdown and the scrambled control were therefore 37.55% and 100%. Of the PRC2 subunits, EZH2 had the most drastic impact on the establishment of HIV-1 latency, and knockdown of this protein resulted in an approximately 70% reduction in the number of silenced proviruses. It is likely that the recruitment of PRC2 to HIV-1 occurs immediately after HIV-1 infection, as we could detect reductions in HIV-1 silent infection following PRC2 shRNA-mediated knockdown as early as 3 days after HIV-1 infection.

As we expected, pretreatment of Jurkat cells with 3-deazaneplanocin A (DZNep), an EZH2 inhibitor, decreased global trimethylation at H3K27 in these cells and the levels of silent proviruses (see Fig. S7). When we quantified the relative silencing of HIV-1 infection in DZNep-treated cells, treatment with 10 μM DZNep also resulted in approximately 80% reductions in the proportions of HIV-1 silent infection in Jurkat T cells.

### The H3K9 silencing machinery is required to establish latency in Jurkat T cells.

A similar approach was used to define the role of H3K9 methylation machinery in the establishment of HIV-1 latency after the knockdown of a series of proteins in the H3K9 methylation machinery (KDM1 [LSD1], EHMT2, and SUV39H1) with shRNAs (see Fig. S6). The most potent effects were obtained with KDM1 (LSD1) shRNA, which led to approximately 65% of the cells having lower levels of H3K9me2-3. However, the effects of knocking down components of the H3K9 methylation machinery were not as pronounced (3-fold change) as that of knocking down PRC2 components (3- to 5-fold). Consistent results were obtained with the small-molecule inhibitors of the H3K9 methylation machinery BIX01294, UNC-0638 (EHMT2 inhibitors), and phenelzine (KDM1 [LSD1] inhibitor) (see Fig. S7 and S8).

10.1128/mBio.00133-17.6 FIG S6  H3K9 methyltransferases and KDM1 (LSD1) are involved in the establishment of HIV-1 latency in Jurkat T cells. (A) Two-dimensional histograms showing the global levels of H3K9me2-3 and HIV-1 infection in the knockdown cells indicated under unstimulated and SAHA-stimulated conditions. The gates for H3K9me2-3 levels were set on the basis of the fluorescence intensity of cells stained only with the mouse IgG secondary antibody labeled with PE-Cy7. (B) FACS analysis of global levels of H3K9me2-3 in EHMT2 (G9a), KDM1 (LSD1), and SUV39H1 knockdown cells. Black, cell counts per quadrant; red, distribution of GFP^+^ cells in high-H3K9me2-3 gate; blue, distribution of GFP^+^ cells in low-H3K9me2-3 gate. (C) Quantification of inducible viruses (percent) in populations of cells having different levels of H3K9me2-3 and relative silencing of HIV-1 in cells expressing shRNAs targeting H3K9 methyltransferases and KDM1 (LSD1). The percentage of inducible viruses in each population resulted from the division of the difference in the normalized numbers of d2EGFP^+^ cells before and after SAHA stimulation by the total number of d2EGFP^+^ cells following SAHA stimulation. As shown, the majority of the inducible viruses were detected within the high-H3K9me2-3 population of cells, while only a small proportion of inducible viruses was present in the low-H3K9me2-3 population. The relative silencing value was analyzed by dividing the normalized total percentage of silent viruses from each knocked-down condition by that from scrambled shRNA treatment. Depletion of EHMT2 (G9a), SUV39H1, or KDM1 (LSD1) resulted in a significant decrease in HIV-1 silent integrations. Download FIG S6, TIF file, 4.2 MB. Copyright © 2017 Nguyen et al. 2017 Nguyen et al. This content is distributed under the terms of the Creative Commons Attribution 4.0 International license.

10.1128/mBio.00133-17.7 FIG S7  Inhibition of EZH2 methyltransferase activity by DZNep or EHMT2 (G9a) by BIX01294 prior to HIV-1 infection reduces silent integrations in Jurkat T cells. Cells were treated overnight with BIX01249 (5 µM), DZNep (5 or 10 µM), or a combination of BIX01249 and DZNEp and then thoroughly washed twice with phosphate-buffered saline. Treated cells were then infected with HIV-1. After 3 days, proviruses were reactivated with SAHA (1 µM) overnight. Flow cytometry intracellular staining was performed to measure the levels of H3K27 trimethylation and H3K9 di- or trimethylation in the treated cells. The gate for FACS analyses of H3K27 trimethylation was based on cells stained with the isotype control antibody (Alexa Fluor 647-conjugated rabbit IgG XP MAb; 2985; Cell Signaling). The gate for H3K9 di- or trimethylation was set on the basis of cells stained only with an anti-mouse IgG1 PE-Cy7-conjugated secondary antibody. HIV-1 infection (d2EGFP expression) was also measured by FACS. (A) FACS analysis of HIV-1 infection and H3K9me2-3 levels in cells pretreated with 5 µM BIX01294, 5 µM DZNEp, or a combination of BIX01294 and DZNEp under unstimulated or SAHA-stimulated conditions. Black, cell counts per quadrant; green, distribution of GFP^+^ cells in the total population. (B) FACS analysis showing H3K9me2-3 levels in Jurkat T cells pretreated with 5 µM BIX01294 overnight. (C) FACS analysis showing H3K27me3 levels in Jurkat T cells pretreated with 5 µM DZNep overnight. (D) Relative silencing of HIV-1 infection in cells pretreated overnight with the inhibitors indicated. Error bars represent the SEM of two independent experiments. Download FIG S7, TIF file, 4.4 MB. Copyright © 2017 Nguyen et al. 2017 Nguyen et al. This content is distributed under the terms of the Creative Commons Attribution 4.0 International license.

10.1128/mBio.00133-17.8 FIG S8  Treatment with the EHMT2 (G9a) inhibitor UNC-0638 or the KDM1 (LSD1) inhibitor phenelzine prior to HIV-1 infection reduces silent integrations in Jurkat T cells. Cells were treated overnight with UNC-0638 (5 µM), phenelzine (250 µM), DZNep (5 µM), or a combination of 5 µM DZNEp with each of above inhibitors and then thoroughly washed twice with phosphate-buffered saline. Treated cells were then infected with HIV-1. After 3 days, proviruses were reactivated with SAHA (1 µM) overnight. Intracellular staining was performed with an anti-H3K9me2-3 antibody to measure the levels of H3K9 di- or trimethylation in the treated cells and then analyzed by FACS. The gate for FACS analyses of H3K9 di- or trimethylation was set on the basis of cells stained only with an anti-mouse IgG1 PE-Cy7 secondary antibody. HIV-1 infection (d2EGFP expression) was also measured by FACS. (A) Two-dimensional histograms measuring HIV-1 infection and H3K9me2-3 levels in cells pretreated with the inhibitors indicated. Black, cell counts per quadrant; green, distribution of GFP^+^ cells in the total population. (B) FACS analysis showing H3K9me2-3 levels in Jurkat T cells pretreated overnight with 5 µM UNC-0638, 250 µM phenelzine, or 5 µM DZNep. (C) Relative silencing of HIV-1 infection in cells pretreated overnight with the inhibitors indicated. Error bars represent the SEM of two independent experiments. Download FIG S8, TIF file, 4.9 MB. Copyright © 2017 Nguyen et al. 2017 Nguyen et al. This content is distributed under the terms of the Creative Commons Attribution 4.0 International license.

### Inhibition of EZH2 or EHMT2 methyltransferase activity reactivates latent HIV-1 in resting T cells cultured *ex vivo*.

To study the role of histone methyltransferases in HIV latency in primary cells, we performed a similar set of experiments by using a polarized primary cell model (31). The model was designed to recapitulate the establishment of HIV-1 latent reservoirs in effector T cells that revert to a resting G0 state to become memory T cells (12). Briefly, naive CD4^+^ cells from healthy donors were polarized into Th17, Th1, and Th2 cells. The polarized cells were infected with VSV-G-pseudotyped HIV-1 and forced into quiescence by culturing in medium containing low concentrations of cytokines.

With this protocol, about 80% of the HIV-1-infected cells became latent after 27 days and maintained high viability (see Fig. S9A). More than 70% of the quiescent cells lacked the cellular proliferation markers Ki-67 and CycD3. pSer175, a marker of activated P-TEFb (32), was also absent from the resting G0 cells. HIV can then be latently reactivated in the vast majority of the cells through TCR activation with Dynabeads Human T-Activator CD3/CD28.

10.1128/mBio.00133-17.9 FIG S9  Reactivation of HIV-1 latent proviruses in Th17, Th1, and Th2 resting memory T cells by GSK343 and EPZ-6438. Naive CD4^+^ T cells were isolated from PBMCs by negative selection. Cells were activated by Dynabeads Human T-Activator CD3/CD28 and polarized into Th1 or Th2 cells. Afterward, cells were infected with VSV-G-pseudotyped Nef^+^ HIV-1. Cells were forced into quiescent conditions by being cultured in medium supplemented with a low concentration of IL-2 (for Th1 and Th2 cells) or IL2 and IL23 (for Th17 cells) for 2 weeks. Reactivation of proviruses was performed by incubating cells for 96 h with GSK343 or EPZ-6438 or by incubating cells overnight with Dynabeads Human T-Activator CD3/CD28. (A) Representative FACS analysis showing the reactivation of HIV-1 latent proviruses with 100 nM GSK343 or EPZ-6438 in Th1 resting memory T cells. (B) Dose-dependent response curves of HIV-1 to GSK343 (black) or EPZ-6438 (red) stimulation in Th1 resting memory T cells. Curves of HIV-1 reactivation corresponding to increasing concentrations of GSK343 on Th17, Th1, or Th2 resting memory T cells. Treatment of GSK343 or EPZ-6438 alone spontaneously reactivates the latent proviruses in all of the subsets of resting memory T cells tested. The levels of HIV-1 reactivation induced by these EZH2 inhibitors are decent compared to that of TCR stimulation. GSK343 is slightly more potent than EPZ-6438 in the reactivation of latent HIV-1. Download FIG S9, TIF file, 23.7 MB. Copyright © 2017 Nguyen et al. 2017 Nguyen et al. This content is distributed under the terms of the Creative Commons Attribution 4.0 International license.

**FIG 4:**
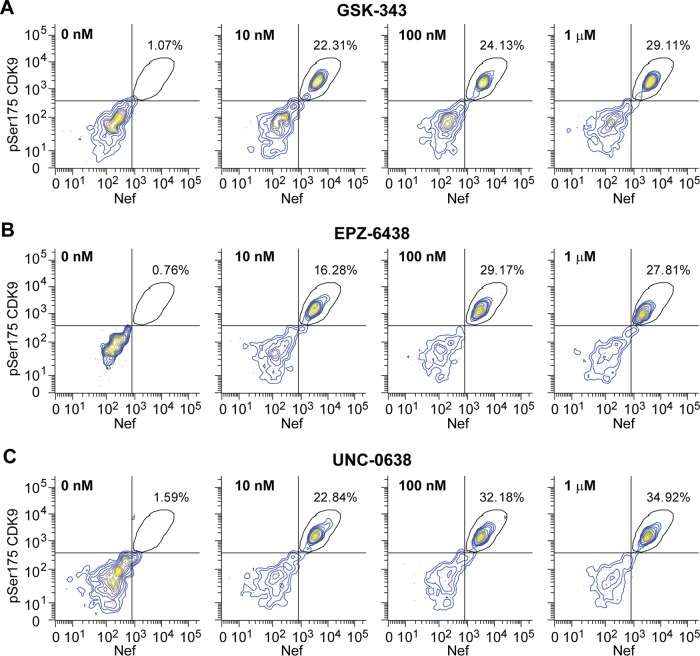
Reactivation of HIV-1 latent proviruses in Th17 resting memory T cells by histone methyltransferase inhibitors. Cells were treated for 96 h with the concentrations of GSK-343 (A), EPZ-6438 (B), or UNC-0638 (C) indicated. Reactivation of latent HIV-1, monitored by Nef expression, and P-TEFb reactivation, monitored by phosphorylated S175 CDK9 levels, were measured by FACS.

Several extremely selective and potent small-molecule inhibitors of EZH2, such as GSK-343 and EPZ-6438, have recently been developed (33, 34). EPZ-6438 is currently being used in clinical trials with patients with B-cell lymphoma (http://clinicaltrials.gov/ct2/show/NCT02601950). Primary cells harboring latent HIV-1 were treated with increasing concentrations of GSK-343 or EPZ-6438 for 4 days. Reactivation of latent proviruses, indicated by Nef expression, and induction of P-TEFb, indicated by enhanced levels of CDK9 phosphorylation at S175 (pSer175-CDK9), were measured by FACS (Fig. 4). As shown in Fig. 4A and B (see also Fig. S9), substantial proviral reactivation (as measured by GFP and Nef expression) was seen in all three polarized cell types with low doses of GSK-343 and EPZ-6438 alone without the need to combine them with other agents. Levels of reactivation of HIV-1 in cells treated with as little as 10 nM GSK-343 or EPZ-6438 were 20 to 25% of the cell population.

To our surprise, we also observed a dose-dependent reactivation of latent proviruses when we treated resting Th17 cells with various concentrations of the H3K9 methyltransferase inhibitor UNC-0638 (Fig. 4C). The maximum level of reactivation following UNC-0638 treatment (35%) was comparable to that achieved with GSK-343 or EPZ-6438, although UNC-0638 was slightly more toxic than GSK-343 or EPZ-6438. These data indicated that unlike what we saw in our T-cell-line model, EHMT2 is also involved in the maintenance of HIV-1 latency in Th17 cells cultured *ex vivo*.

### Histone methyltransferases are required for establishment of latency in primary cells.

**FIG 5:**
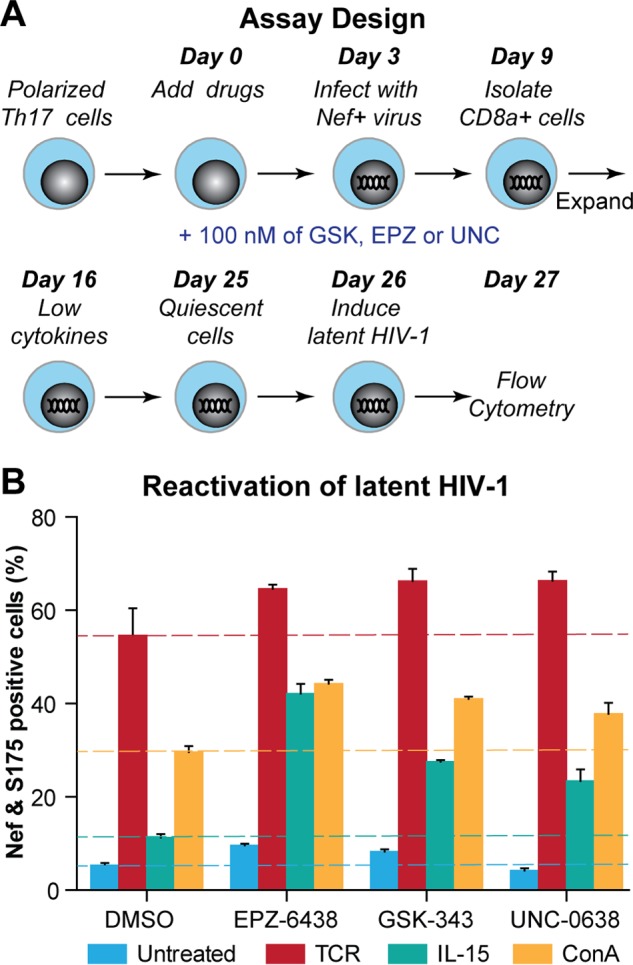
EZH2 and EHMT2 are required for the establishment of latent HIV-1 in primary Th17 cells. (A) Experimental design. (B) Reactivation of latent proviruses by Dynabeads Human T-Activator CD3/CD28, IL-15 (50 ng/ml), or ConA (5 µg/ml) in cells pretreated with DMSO, 100 nM GSK-343, EPZ-6438, or UNC-0638.

We next investigated the role of EZH2 and EHMT2 in the establishment of HIV-1 latency in the Th17 cell model. Th17 cells were treated with GSK-343, EPZ-6438, or UNC-0638 (100 nM) for 72 h and then infected with HIV-1 (Fig. 5A). HIV-1-infected cells were isolated and cultured under low-cytokine conditions to force entry into quiescence and silence the proviruses. The inhibitors were replenished every 72 h. On day 26, the quiescent cells were washed to remove the drugs and latent proviruses were induced with three different stimulators, Dynabeads Human T-Activator CD3/CD28, interleukin-15 (IL-15), or concanavalin A (ConA). Reactivated proviruses were detected by FACS measuring Nef and pSer175-CDK9 levels.

Following dimethyl sulfoxide (DMSO) treatment, 5% of the activated proviruses were detected, while approximately 8%, 10%, and 4% of the activated proviruses were detected in cells treated with GSK-343, EPZ-6438, or UNC-0638, respectively (Fig. 5B). These values indicated that there were fewer proviruses progressing into latency in cells treated with EZH2 inhibitors than in those treated with DMSO or the EHMT2 inhibitor. When IL-15 and ConA, which are relatively weak activators of latent proviruses, were added, enhanced activation was observed (Fig. 5B). For example, 10% of the latent proviruses were reactivated from cells pretreated with DMSO and induced with 50 ng/ml IL-15, whereas 27% (GSK-343), 40% (EPZ-6438), and 22% (UNC-0638) were induced from cells pretreated with epigenetic modulators. Reactivation of latent proviruses by TCR stimulation was also enhanced in cells pretreated with EZH2 or the EHMT2 inhibitor compared to that in DMSO-pretreated cells, although the magnitude of this effect was limited since virtually all of the latent proviruses were activated under these conditions in cells not treated with drugs.

### Epigenetic modulators reactivate latent HIV-1 from well-suppressed patients.

**FIG 6:**
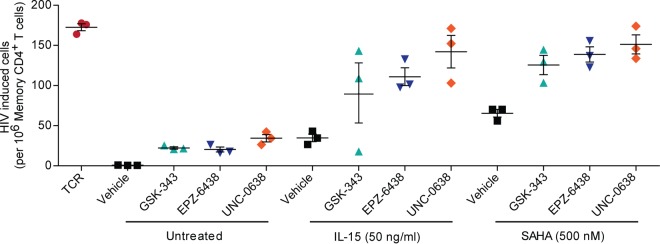
Induction of latent HIV-1 in CD4^+^ memory T cells from well-suppressed patients by histone methyltransferase inhibitors. CD4^+^ memory T cells isolated from HIV-1-infected patients were treated for 72 h with 100 nM GSK-343, EPZ-6438, or UNC-0638 in the presence or absence of IL-15 (50 ng/ml) or SAHA (500 nM) for 16 h. The levels of spliced HIV-1 *env* mRNA were measured by the EDITS next-generation sequencing assay and are presented as numbers of cells containing reactivated HIV-1 per 10^6^ cells.

Our data on Th17 primary cells indicated that GSK-343, EPZ-6438, and UNC-0638 were potent LRAs. Therefore, we investigated whether these compounds could reactivate the transcription of latent proviruses in HIV-1-infected patients undergoing HAART and how they reactivated latent proviruses when used in combination with other LRAs. Memory CD4^+^ T cells were isolated from three different donors and treated with 100 nM GSK-343, EPZ-6438, or UNC-0638 for 72 h. Cells were then further treated overnight with IL-15 (50 ng/ml) or SAHA (500 nM). Reactivation of latent provirus transcription was then measured with the EDITS assay, which measures the abundance of singly spliced *env* mRNAs by next-generation sequencing. Levels of latent provirus reactivation were calculated as the number of cells harboring HIV-1 *env* mRNAs per million cells. Cell numbers were based on a standard curve prepared from sorted HIV-expressing cells (Fig. 6). TCR stimulation, which is the most potent known stimulator of latently infected patient cells, resulted in reactivation of HIV-1 in 170/10^6^ cells, while the epigenetic modulators led to reactivation of HIV-1 in 20 to 35/10^6^ cells. The most effective activator was UNC-0638, which caused reactivation of HIV-1 in 35/10^6^ cells.

Importantly, all of the compounds exhibited synergistic reactivation effects when combined with IL-15 and SAHA in all three donors. For instance, single treatment with GSK-343 and IL-15 resulted in reactivation of latent HIV-1 in 22 and 34/10^6^ cells, respectively. However, combined treatment with GSK-343 and IL-15 led to reactivation of HIV-1 in 125/10^6^ cells, which is close to the levels achieved with TCR stimulation. Similar values were measured following treatment with the other LRA combinations, ranging from 100 to 150/10^6^ cells. Thus, GSK-343, EPZ-6438, and UNC-0638 induce reactivation of latent HIV-1 in central memory CD4^+^ T cells recovered from well-suppressed patients. Furthermore, each one of these inhibitor acts synergistically with IL-15 or SAHA to effectively reactivate latent HIV-1.

## DISCUSSION

### The H3K27 and H3K9 methylation machineries.

Methylation of histone H3 at lysines 9 and 27 is strongly correlated with the transcriptional repression of genes and formation of heterochromatin (35) and is controlled primarily by three HKMT complexes, PRC2, CtBP (EHMT2/GLP), and SUV39H1. The PRC2 core complex, which includes four proteins (EZH1/EZH2, SUZ12, EED, and RbAp46/48) (29), is responsible for di- and trimethylation of H3K27, although the PRC2-EZH1 complex has lower methylase activity than the PRC2-EZH2 complex. The recently identified PRC2 auxiliary factors AEBP2, PCLs, and JARID2 (29, 36, 37) are believed to play a role in the fine-tuning of PRC2 activity or modulation of the recruitment of PRC2 to its target chromatin (36, 38). SUV39H1 and EHMT2 belong to the SUV39H subgroup of SET domain-containing molecules and catalyze H3K9 mono-, di-, and trimethylation (39). SUV39H1 is required for the formation of pericentromeric heterochromatin (40), whereas EHMT2 forms a stoichiometric heterodimer with GLP, an EHMT2 like protein that dominantly catalyzes mono- and dimethylation of H3K9 at euchromatin (40). In addition to their primary function of methylating histones, these enzymes may also target transcription factors, such as STAT3, p53, GATA4, and NF-κB (41, 42), at the proviral promoter, expanding their potential effects on transcription.

### Only PRC2 is essential for maintenance of HIV-1 latency in Jurkat T cells.

We have previously shown that EZH2 is required to maintain proviral latency (24). In the present study, we expanded these results by knocking down each of the subunits of PRC2 (EZH2, EED, SUZ12, and RBBP7) and demonstrating proviral reactivation (see Fig. S1). Similarly, our unbiased shRNA library screening identified most of the subunits of PRC2 as strong hits (Table 1). These observations are consistent with other findings showing that EED and SUZ12 are also crucial for the methyltransferase activity of the enzymatic subunit EZH2 (36, 37). ChIP assays showed that all of the core subunits of PRC2 are enriched at regions surrounding the HIV-1 promoter and displaced when HIV-1 transcription is reactivated (see Fig. S1D). Thus, the entire PRC2 complex is recruited to the HIV-1 promoter to impose H3K27me3 restriction and suppress HIV-1 transcription.

Depletion of components of the H3K9 methylation machinery (EHMT2, SUV39H1, and KDM1 [LSD1]) did not measurably reverse HIV-1 latency in Jurkat cells, even though these factors are also present at the provirus promoter (see Fig. S2). Similarly, treatment of latently HIV-1-infected cells with an EHMT2 inhibitor (BIX01294) or a potent SUV39H1 inhibitor (chaetocin) did not effectively reactivate proviruses (24).

The results imply that H3K27me3 is dominant over H3K9 methylation in maintaining HIV-1 latency in the cell lines tested. The persistence of PRC2 at the promoter under conditions in which EHMT2 is removed may provide an explanation for the greater repression imposed by PRC2. Removal of EZH2 by CRISPR-mediated gene disruption results in the loss of both EZH2 and EHMT2 from the LTR, whereas depletion of EHMT2 is more selective (Fig. 2).

### Both EHMT2 and EZH2 are required to establish HIV-1 latency.

Depletion of each of the subunits of PRC2 in Jurkat T cells also resulted in reduced silencing of HIV-1. EZH2 depletion had the greatest impact, presumably because of its enzymatic activity; however, components of the H3K9 methylation machinery (KDM1 [LSD1], EHMT2, and SUV39H1) were also necessary for establishment of viral silencing (Fig. 3D; see Fig. S6). Similar results were obtained with inhibitors to block EZH2 and EHMT2 activity, suggesting that the two machineries function in conjunction to establish HIV-1 silencing.

We also detected a role for KDM1 (LSD1) in the establishment, but not the maintenance, of HIV-1 latency in T-cell lines, implying that it acts as a repressor of HIV-1 transcription. This is in agreement with a previous study showing that KDM1 (LSD1) represses HIV-1 transcription in microglial cells by cooperating with CTIP2 to recruit HDAC and SUV39H1 to the HIV-1 promoter (43).

### Multiple histone methyltransferases are required for the establishment and maintenance of HIV-1 latency in resting memory T cells.

Pharmacological inhibition of EZH2 by GSK-343 and EPZ-6438 was able to induce latent proviruses in primary Th1, Th2, or Th17 resting memory cells infected *ex vivo*. In contrast to the Jurkat cell system, where EZH2 inhibition led to relatively modest latency reversal, HIV-1 in our primary T cells was substantially reactivated by GSK-343 or EPZ-6438 (Fig. 4; see Fig. S9). Furthermore, reactivation experiments we performed with memory CD4^+^ T cells from HIV-1-infected donors also show that GSK-343 and EPZ-6438 can induce the transcription of latent HIV-1 (Fig. 5).

In contrast to what we observed in Jurkat cells, reactivation experiments with UNC-0638 on latently HIV-1-infected primary Th17 cells cultured *ex vivo* (Fig. 4) and memory CD4^+^ T cells from HIV-1-infected donors (Fig. 6) demonstrated the involvement of EHMT2 in the maintenance of HIV-1 latency in primary cells.

Our results obtained with latently HIV-1-infected primary T cells are somewhat inconsistent with the findings of Tripathy et al. (23). Those authors used the Lewin model, in which resting cells were activated by CCL19 and then allowed to revert to quiescence for a limited period of time. Under these conditions, GSK-343 was an ineffective inducer of HIV, although it did show enhanced reactivation with SAHA. It seems likely that the epigenetic transcriptional block induced by EHMT2 and PRC2 on HIV-1 is gradually built up, and additional layers of epigenetic restrictions, including DNA methylation (44, 45), may be imposed over time.

Treatment of cells with EZH2 or EHMT2 inhibitors during the establishment of HIV-1 latency in Th17 cells slightly reduces the yield of latent proviruses and highly sensitizes them to exogenous stimulators such as IL-15, ConA, or TCR (Fig. 5). Thus, inhibition of either histone methyltransferase reduces the efficacy of the epigenetic restrictions imposed on HIV-1 as it progresses toward latency in primary T cells. The partial silencing that can be achieved by single histone methyltransferases in this system is perhaps due to additional blocks to HIV transcription also imposed in quiescent T cells by the lack of P-TEFb and sequestration of the transcription initiation factors NFAT and NF-κB (16).

### Epigenetic modulators are effective LRAs in patient cells.

In this report, we have described for the first time the use of EZH2 and EHMT2 histone methyltransferase inhibitors as HIV-1 LRAs in memory CD4^+^ T cells isolated from HIV-1-infected donors receiving HAART. Numerous studies indicate that combinations of different LRAs are more effective than single agents in reactivating latent HIV-1 reservoirs (22, 46). This is also true of the histone methyltransferase inhibitors, which exhibit synergistic effects when used combinatorially with other agents, such as IL-15 or SAHA. Tripathy et al. (23) also observed synergy between SAHA and GSK-343 in proviral reactivation in primary cells.

In conclusion, our findings indicate that PRC2 and EHMT2 both play essential roles in the establishment and maintenance of HIV-1 latency in resting memory cells infected *ex vivo* and *in vivo*. Small molecules inhibiting histone H3 lysine methyltransferases, such as GSK-343, EZP-6438, or UNC-0638, may therefore find a role as part of a latency-reversing regimen for HIV eradication.

## MATERIALS AND METHODS

### Cell lines and cell culture reagents.

E4, a latently HIV-1-infected cell line, was used (24). Cells were cultured in HyClone RPMI medium with l-glutamine, 10% fetal bovine serum (FBS), penicillin (100 IU/ml), and streptomycin (100 µg/ml) in 5% CO_2_ at 37°C. Primary T cells were cultured in RPMI medium supplemented with 10% FBS, primocin, and 25 mM HEPES (pH 7.2). Cell viability after treatment with drugs was measured by propidium iodide staining.

### VSV-G-pseudotyped HIV-1 production.

VSV-G-pseudotyped HIV-1 was produced as previously described, with the d2EGFP-Nef-pHR' vector (which expresses Nef and d2EGFP), as well as the pdR8.91 and VSV-G vectors (28). Jurkat cells were infected with HIV-1 by spinoculation with viruses at 3,480 rpm for 1.5 h at room temperature.

### CRISPR-Cas9, shRNA constructs, and infections.

Genomic RNA targeting EZH2 (TGAGCTCATTGCGCGGGACT), Jarid2 (GGATTCCGTGGTCAGAAGAA), or EHMT2 (TTCCCCATGCCCTCGCATCC) was cloned into the lentiCRISPR v2 plasmid expressing mCherry (47). lentiCRISPR v2 was a gift from Feng Zhang (Addgene plasmid catalog no. 52961). shRNA sequences targeting different subunits of PRC2 proteins identified from the library screening were cloned into the retroviral pWKC1 backbone (which expresses an mCherry reporter gene) or pSIREN-retroQ (631526; Clontech). The following shRNA sequences were used: scrambled, TTGATGCACTTACTAGATTAC; EZH2, CCCAACATAGATGGACCAAAT; EED, clone ID TRCN0000021206 (Open Biosystems); SUZ12, CCACAAGAAATGGAAGTAGAT; AEBP2, GCACCAAAGTTGGTCTTGAAA; RBBP7, CCTCCAGAACTCCTGTTTATT; KDM1, LSD1; GCTACATCTTACCTTAGTCAT; EHMT2, CCTCTTCGACTTAGACAACAA; SUV39H1, CCTCGGTATCTCTAAGAGGAA.

### Western blotting.

Anti-EZH2 (17-662; Millipore), anti-EED (sc-133537; Santa-Cruz), anti-SUZ12 (17-661; Millipore), anti-Jarid2 (AB192252; Abcam, Inc.), anti-EHMT2 (3356S; Cell Signaling), anti-AEBP2 (AB107892; Abcam, Inc.), anti-RBBP7 (6882S; Cell Signaling), anti-glyceraldehyde-3-phosphate dehydrogenase (sc-47724; Santa-Cruz), anti-β-actin (Santa-Cruz), anti-Spt5 (Santa-Cruz Biotechnologies), anti-histone H3 (AB1791; Abcam, Inc.), anti-histone H3K27me3 (AB6002; Abcam, Inc.), and anti-dimethyl histone H3 (Lys9) (AB1220; Abcam, Inc.) antibodies were used for Western blotting, which was performed as described previously (24). Fifty micrograms of total cell lysate or nuclear extract was loaded.

### ChIP-qPCR analysis.

ChIP was performed as previously described (24), with the Pierce agarose ChIP kit (Thermo Scientific). For ChIP, anti-RNAP II (17-672 [Millipore] or sc-899 [Santa Cruz]), anti-EZH2 (17-662; Millipore), anti-EED (17-10034; Millipore), anti-SUZ12 (17-661; Millipore), anti-Jarid2 (AB192252; Abcam, Inc.), anti-histone H3 (AB1791; Abcam, Inc.), anti-histone H3K27me3 (AB6002; Abcam, Inc.), anti-EHMT2 (3356S; Cell Signaling), anti-SUV39H1 (8729S; Cell Signaling), anti-KDM1 (LSD1) (2139S; Cell Signaling), anti-trimethyl histone H3 (Lys9) (AB8898, Abcam, Inc.), and anti-dimethyl histone H3 (Lys9) (AB1220; Abcam, Inc.) antibodies were used. The percentage-of-input method was used to calculate the enrichment of proteins in specific regions of the HIV-1 genome.

### Flow cytometry intracellular staining.

Cells were fixed with methanol-free formaldehyde (4%) at room temperature for 15 min. Permeabilization of cell membrane was performed in BD Perm/Wash buffer (51-2091 KZ; BD) for 5 min. Cells were stained with Di/TriMethyl histone H3 (Lys9) mouse monoclonal antibody (MAb) (1:400 dilution; 5327; Cell Signaling) for 30 min and then with anti-mouse IgG1 phycoerythrin (PE)-Cy7-conjugated secondary antibody (1:500 dilution; 25-4015; eBioscience) for 20 min. Staining of cells with Alexa Fluor 647-conjugated trimethyl histone H3 (Lys27) (1:750 dilution; 12158; Cell Signaling) was performed for 30 min. An Alexa Fluor 647-conjugated rabbit IgG XP MAb (1:750 dilution; 2985; Cell Signaling) was used as an isotype control. The gate for FACS analyses of H3K9 di- or trimethylation was set on the basis of cells stained only with the anti-mouse IgG1 PE-Cy7-conjugated secondary antibody, and that of H3K27 trimethylation was based on cells stained with the isotype control.

### Production of latently HIV-1-infected primary cells and virus reactivation.

Naive CD4^+^ T cells were negatively isolated from peripheral blood mononuclear cells (PBMCs) with the Human Naive CD4^+^ T Cell Enrichment kit (19155RF; Stem Cell). Cells were cultured in medium supplemented with Dynabeads Human T-Activator CD3/CD28 (25 µl/10^6^ cells), transforming growth factor beta (TGF-β; 5 µg/ml), and anti-IL-4 antibody (500-M04; PeproTech) (10 µg/ml) for 6 days to polarize cells into Th1 cells. To polarize cells into Th2 cells, the anti-IL-4 antibody was replaced with an anti-gamma interferon (IFN-γ) antibody (500-M90; PeproTech) (10 µg/ml). Cytokine antibodies were used to polarize cells into Th17 cells as follows: anti-TGF-β antibody, 5 µg/ml; anti-IL-4 antibody, 10 µg/ml; anti-IFN-γ antibody, 10 µg/ml; anti-IL-1β antibody, 10 µg/ml; anti-IL-6 antibody, 30 µg/ml; anti-IL-23 antibody, 50 µg/ml. Afterward, cells were infected with VSV-G-pseudotyped HIV-1 Nef^+^ virus, which expresses CD8a-d2EGFP. HIV-1-infected cells were purified with an anti-CD8a selection kit (Stem Cell catalog no. 18953). Cells were forced into quiescence by being cultured in medium supplemented with a low concentration of IL-2 (15 IU/ml) (for Th1 and Th2 cells) or low concentrations of IL-2 (15 IU/ml) and IL-23 (12.5 µg/ml) (for Th17 cells) for 2 weeks. Reactivation of proviruses were performed by incubating cells overnight with Dynabeads Human T-Activator CD3/CD28 (25 µl/10^6^ cells).

### Treatment of latently HIV-1-infected primary cells with inhibitors.

Cells were treated with increasing concentrations of GSK-343, EPZ-6438, or UNC-0638 for 96 h. Cell viability was measured by propidium iodide staining. Reactivation of latent provirus, indicated by Nef and pSer175-CDK9 levels, was measured by FACS as described previously (32).

### RNA induction (EDITS) assay.

Memory CD4^+^ T cells were isolated from three different HIV-1-infected donors who were receiving HAART and had undetectable levels of viral RNA with the EasySep Human Memory CD4^+^ T-cell Enrichment kit (Stem Cell catalog no. 19157). One million cells were treated with 100 nM GSK-343, EPZ-6438, or UNC-0638 for 72 h. Cells were left untreated or further treated with IL-15 (50 ng/ml) or SAHA (500 nM) overnight. Total RNA was isolated and subjected to reverse transcription (RT)-PCR with primers that specifically amplified singly spliced *env* mRNA of HIV-1. Next-generation sequencing of RT-PCR products was performed to measure the abundance of *env* mRNAs. The number of reads was converted into the equivalent number of cells harboring HIV-1 per 10^6^ cells by using a standard curve prepared from HIV-infected cells sorted by flow cytometry.
