# Supplementary figures and images for: Multiple Histone Lysine Methyltransferases Are Required for the Establishment and Maintenance of HIV-1 Latency
Source: mBio. 2017 Feb 28;8(1):e00133-17. doi: 10.1128/mBio.00133-17 (PMC5347344; doi:10.1128/mBio.00133-17)

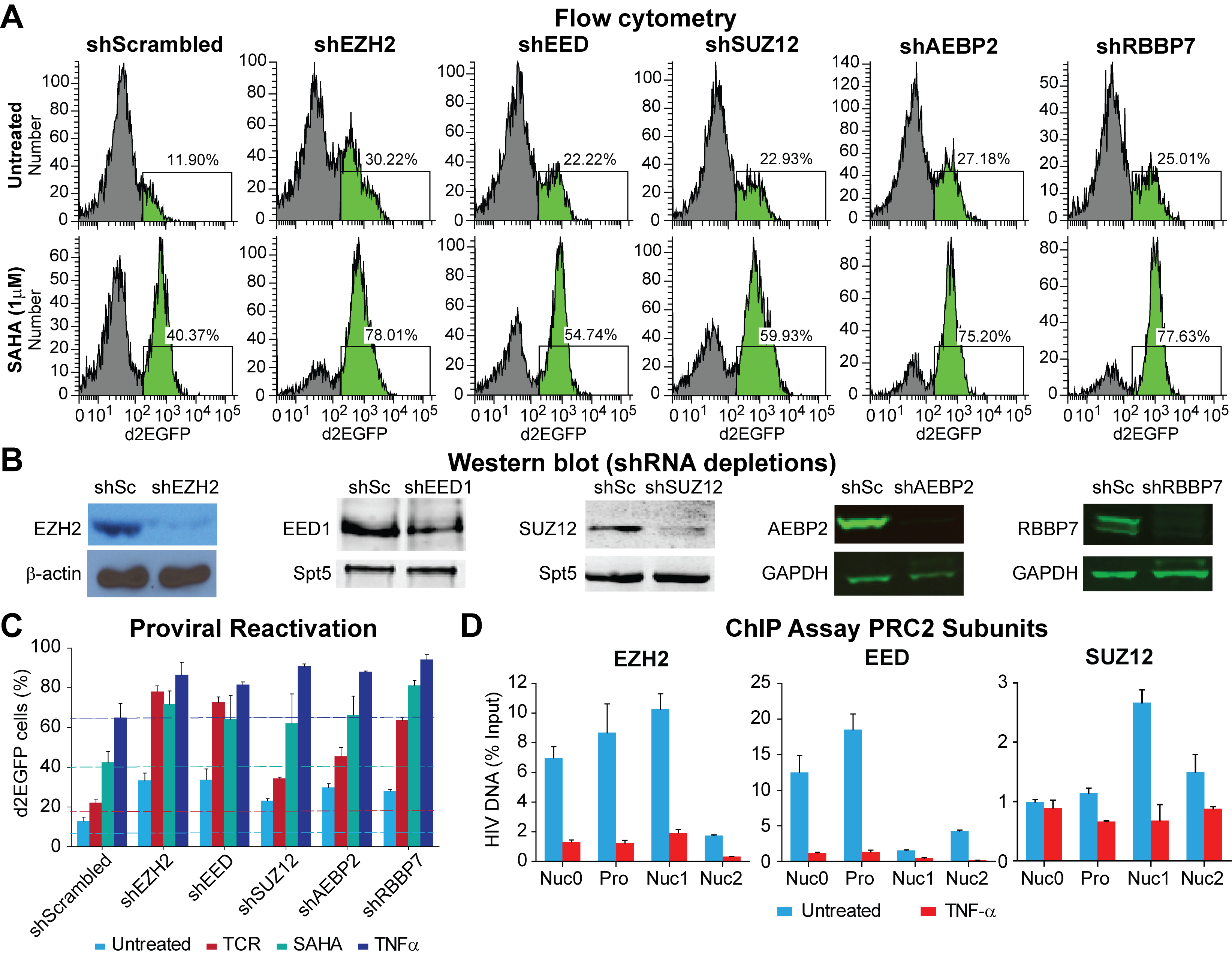

Supplement: FIG S1 [file mbo001173206sf1.tif]

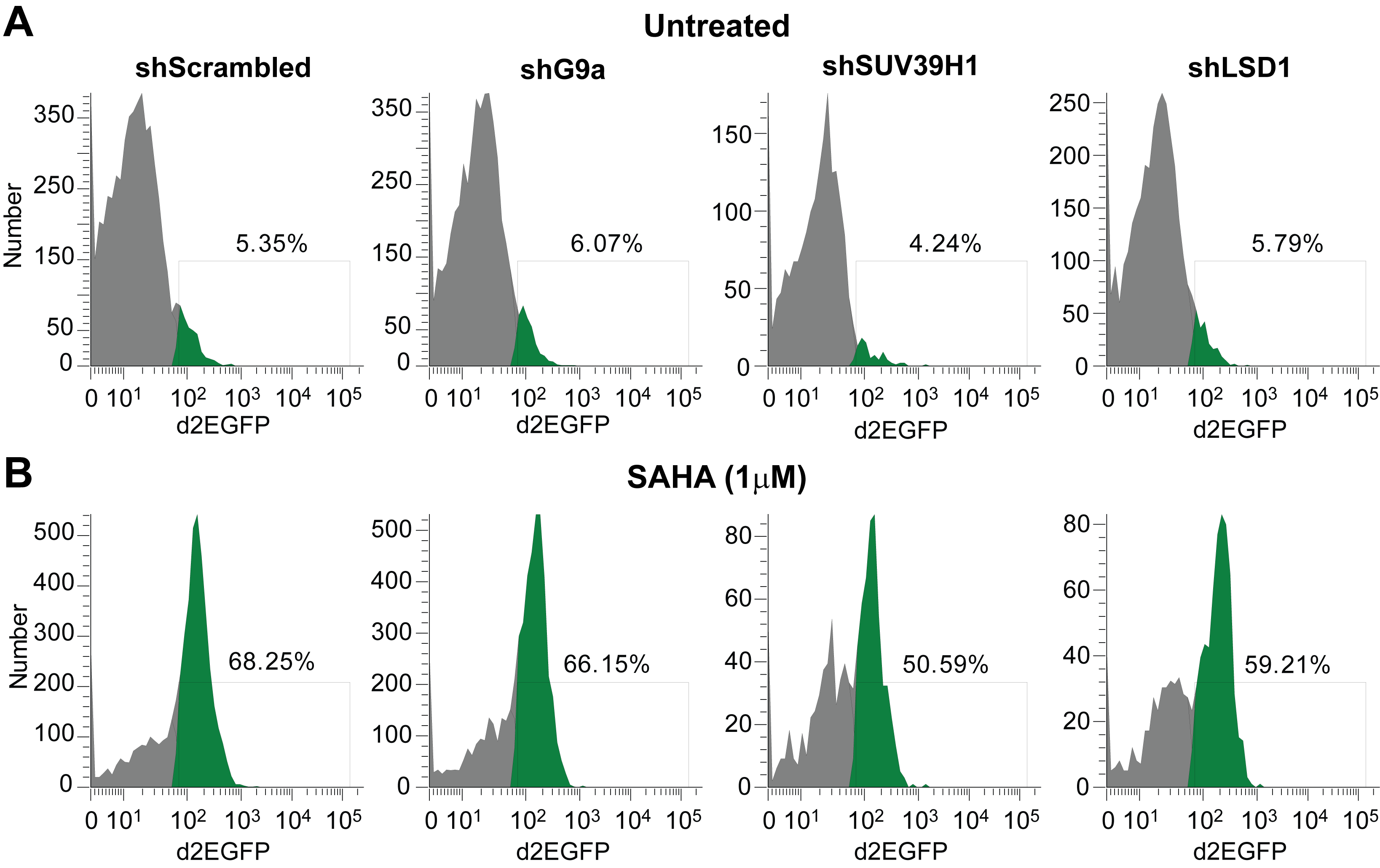

Supplement: FIG S2 [file mbo001173206sf2.tif]

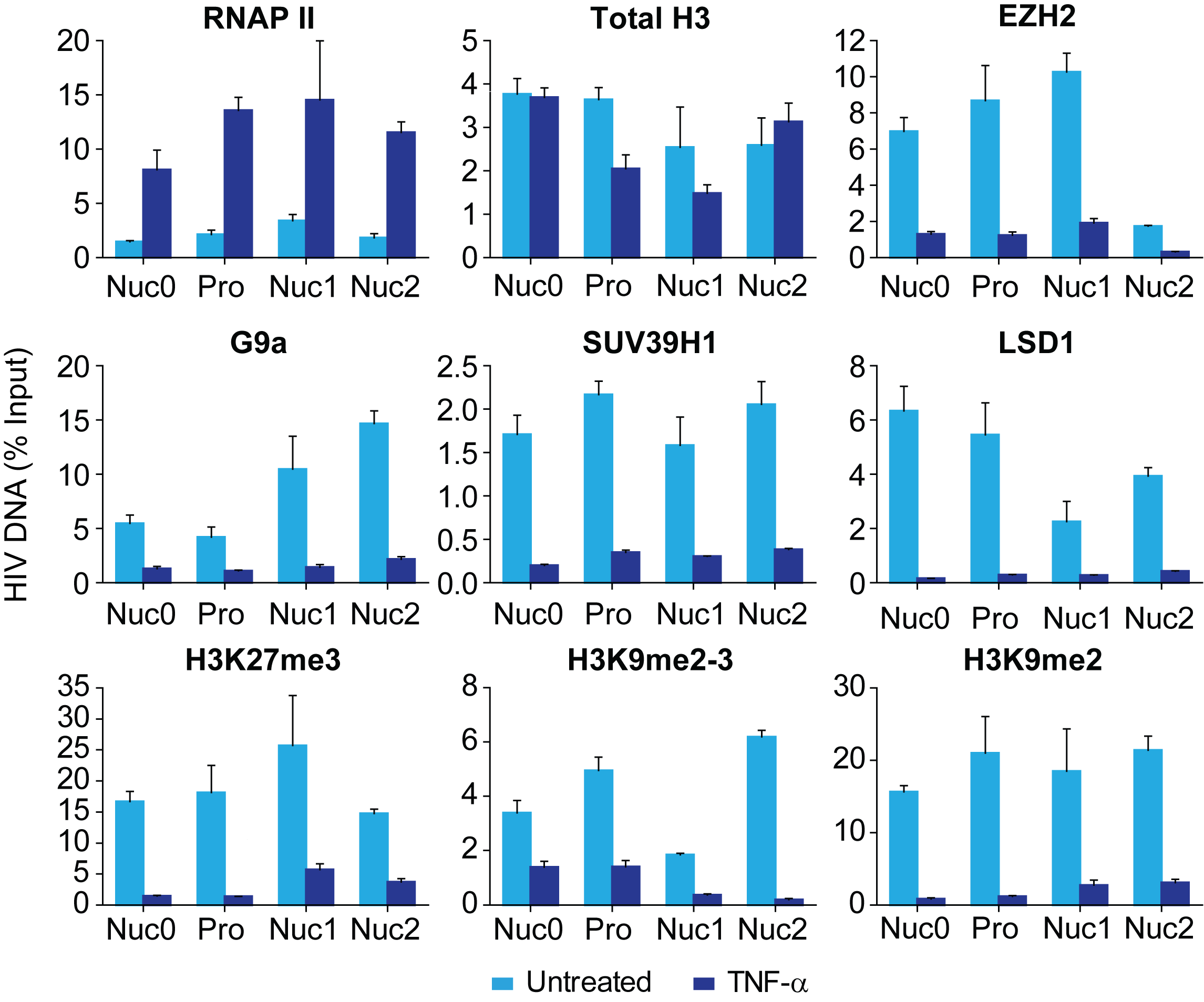

Supplement: FIG S3 [file mbo001173206sf3.tif]

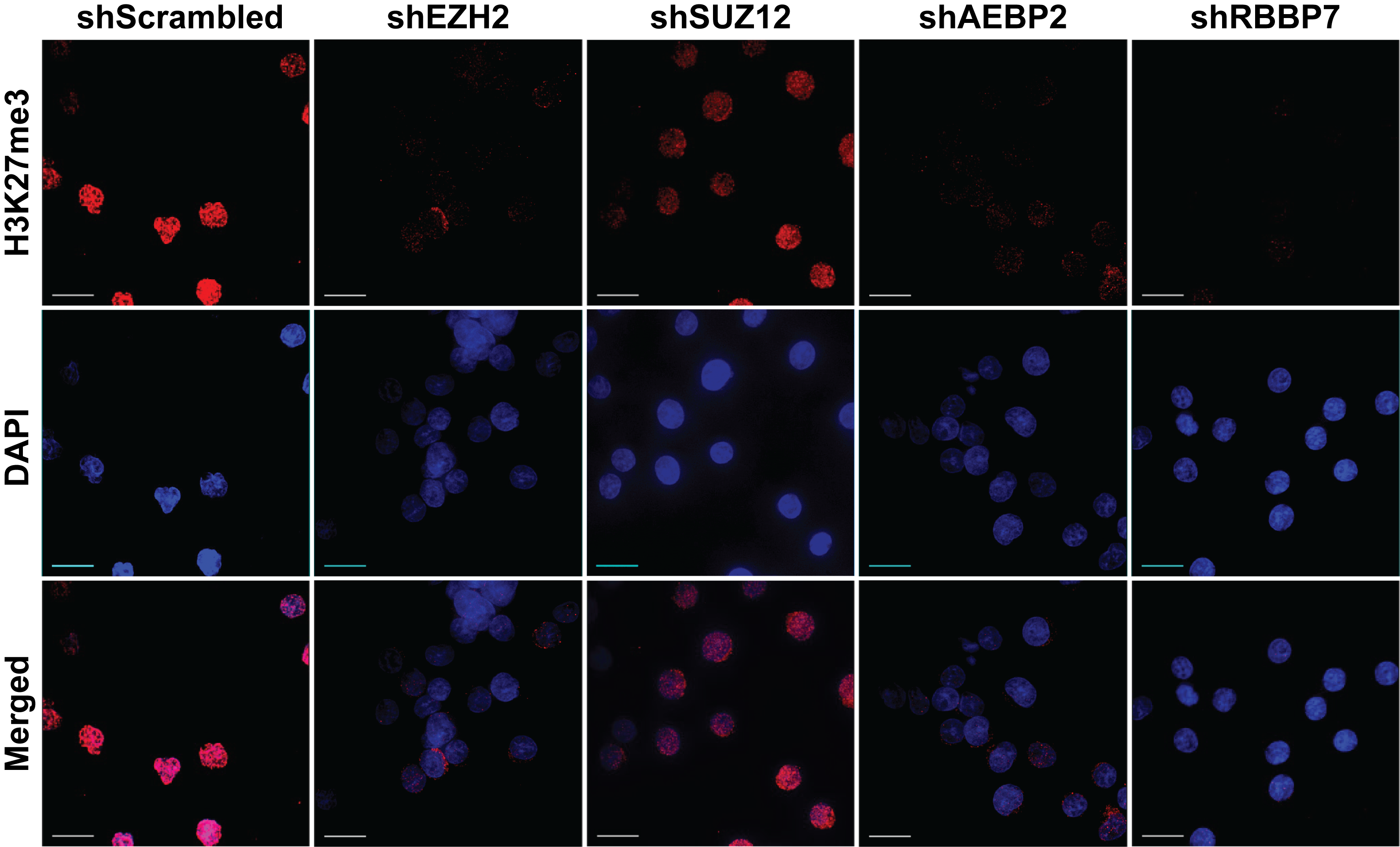

Supplement: FIG S4 [file mbo001173206sf4.tif]

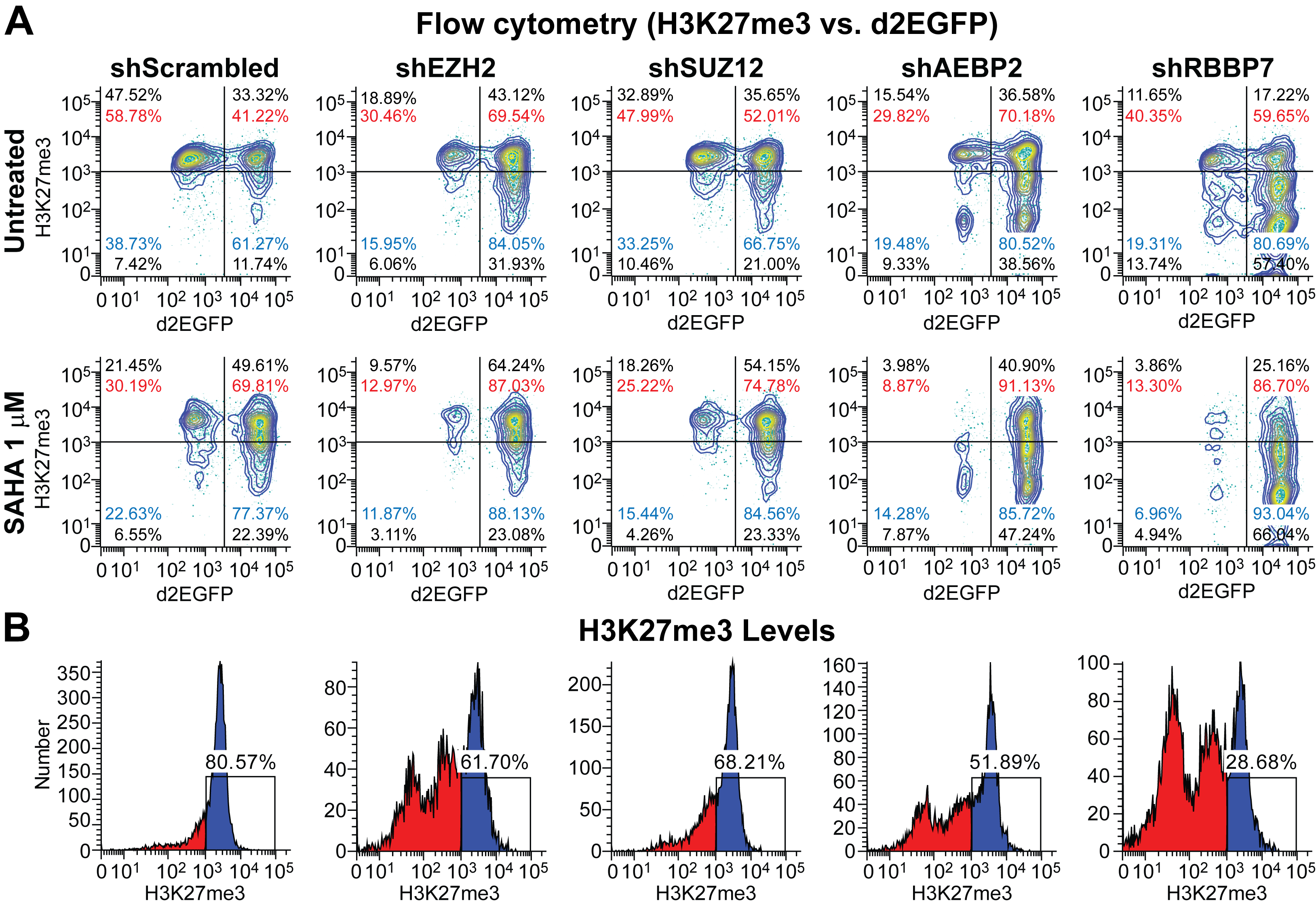

Supplement: FIG S5 [file mbo001173206sf5.tif]

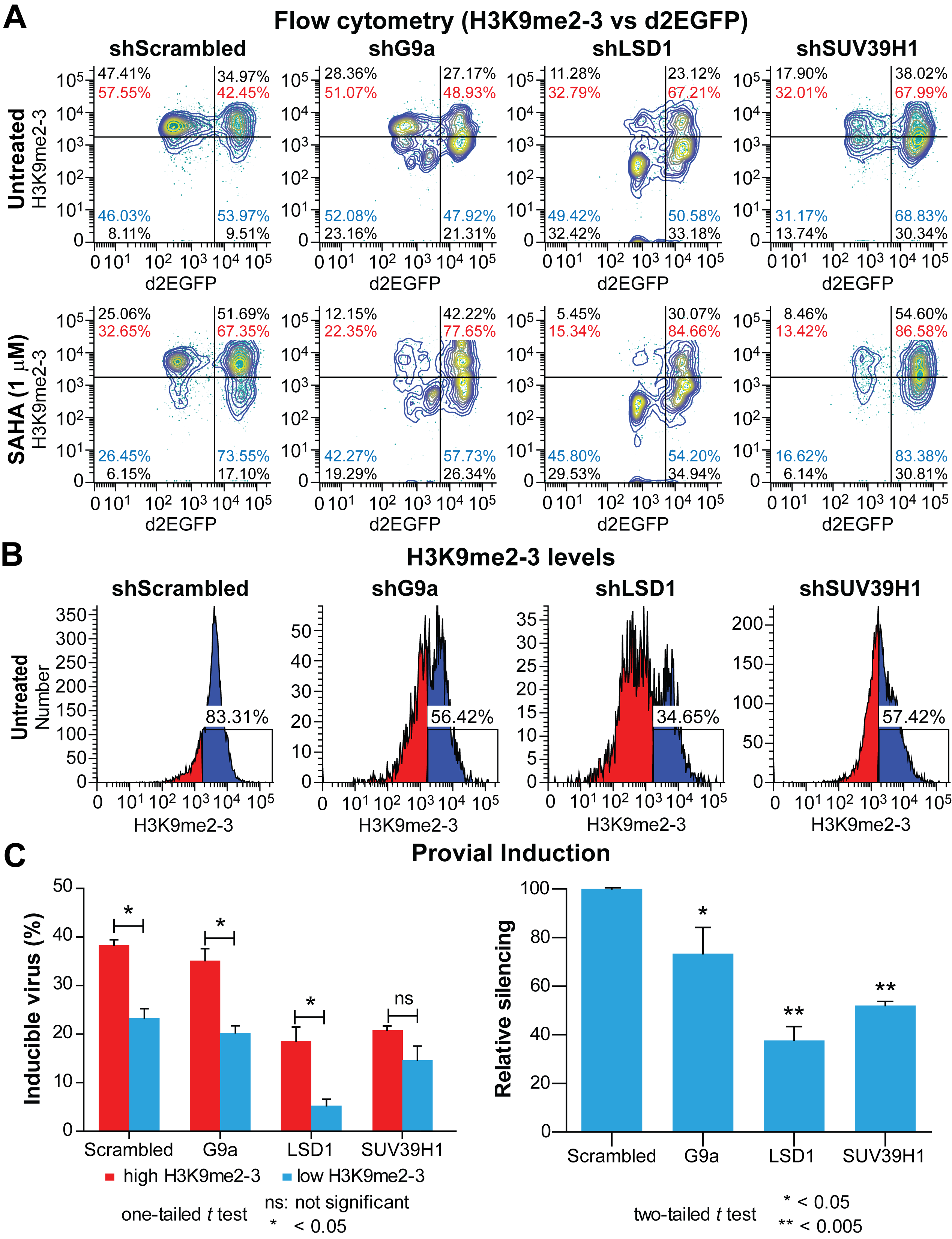

Supplement: FIG S6 [file mbo001173206sf6.tif]

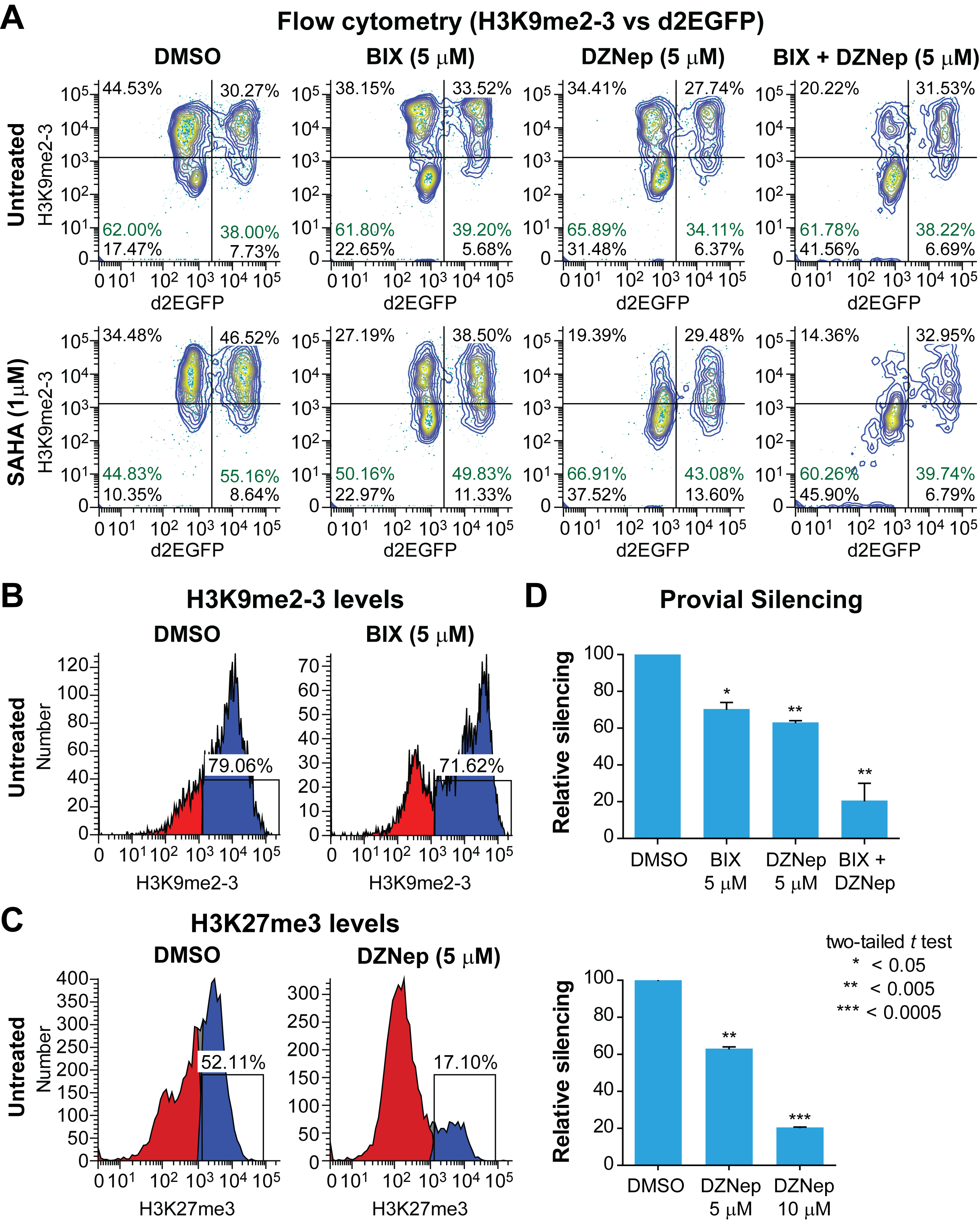

Supplement: FIG S7 [file mbo001173206sf7.tif]

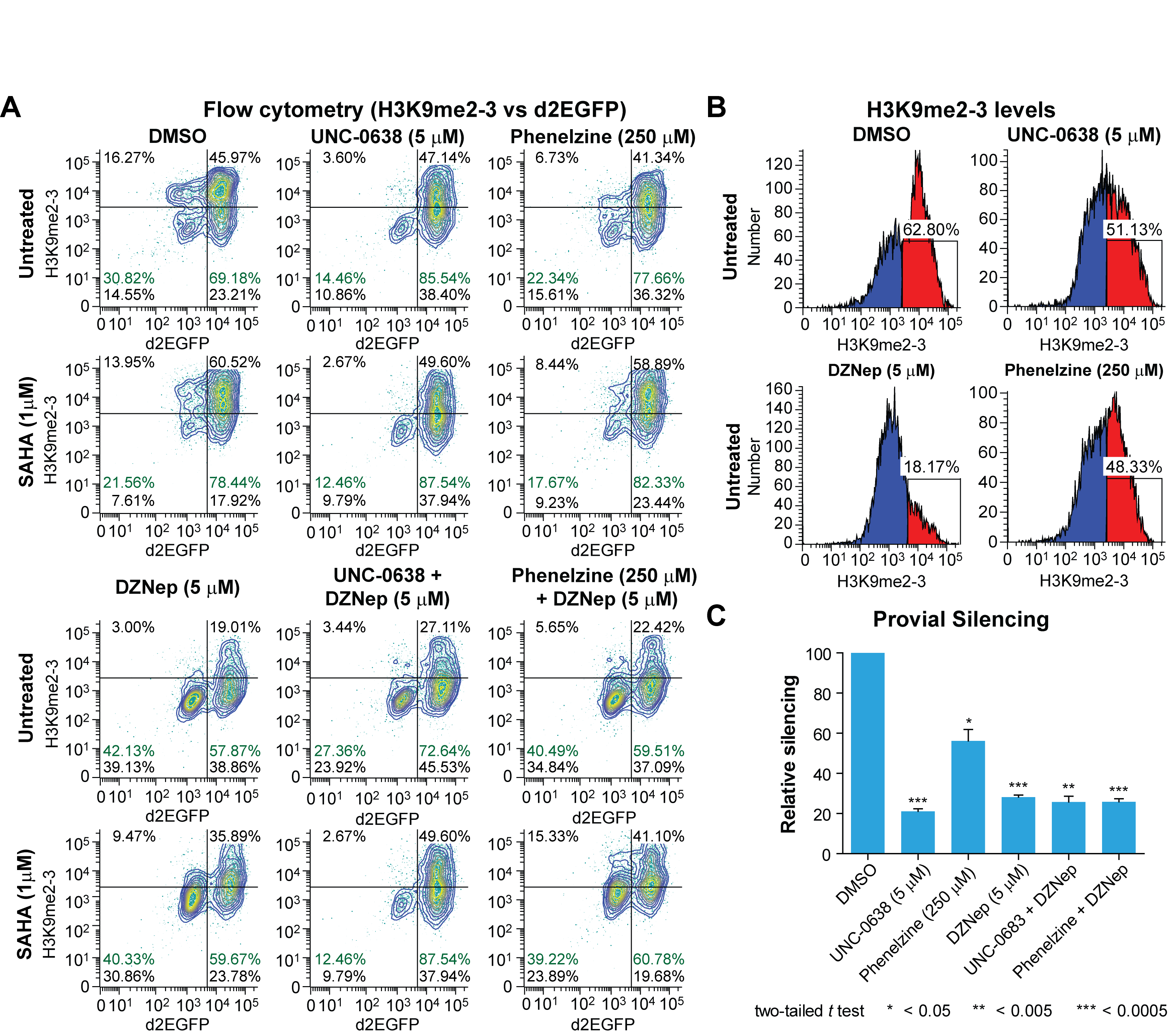

Supplement: FIG S8 [file mbo001173206sf8.tif]

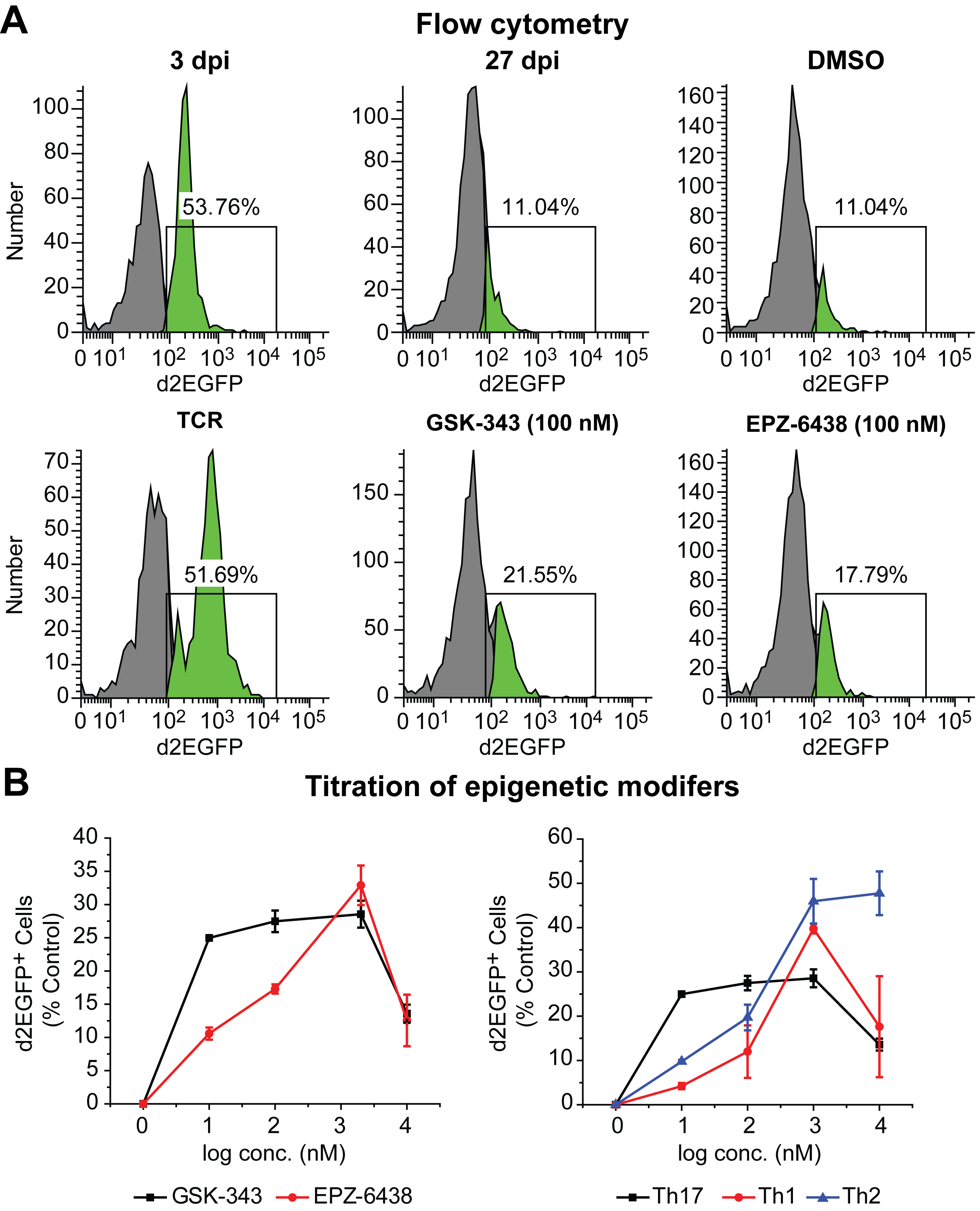

Supplement: FIG S9 [file mbo001173206sf9.tif]
